# The acute effects of alcohol on state rumination in the laboratory

**DOI:** 10.1007/s00213-021-05802-1

**Published:** 2021-02-26

**Authors:** O. Merve Mollaahmetoglu, Edward Palmer, Emily Maschauer, Melissa C. Nolan, Tobias Stevens, Molly Carlyle, Lorna Hardy, Edward R. Watkins, Celia J. A. Morgan

**Affiliations:** 1 Psychopharmacology and Addiction Research Centre (PARC), University of Exeter, Washington Singer Building, Perry Road, Exeter, EX4 4QG UK; 2 School of Psychology, University of Queensland, St. Lucia, QLD Australia; 3 SMART Lab, University of Exeter, Washington Singer Building, Perry Road, Exeter, EX4 4QG UK

**Keywords:** Alcohol use disorders, Rumination, Negative affect, Depression

## Abstract

**Rationale:**

Rumination is a repetitive, negative, self-focused thinking style associated with various forms of psychopathology. Recent studies suggest that rumination increases craving for alcohol and predicts harmful drinking and alcohol-related problems. However, the acute effects of alcohol on rumination have not been previously studied. It is proposed that alcohol may reduce ruminative thinking through decreasing negative mood.

**Objectives:**

In the present study, we aimed to test the previously unexplored effects of acute alcohol consumption on rumination in a hazardous drinking population.

**Methods:**

We conducted a randomised placebo-controlled laboratory study to examine the effect of low (0.4 g kg^−1^) and high doses (0.8 g kg^−1^) of alcohol on state rumination compared to placebo. Participants completed a rumination induction task prior to receiving drinks. We then measured state rumination and mood at repeated time points; 30 min, 60 min and 90 min post-drinks consumption.

**Results:**

We found a significant decrease in state rumination in the low-dose alcohol group compared to placebo at 30 min post-alcohol consumption, but no difference was observed between the high-dose alcohol and placebo groups. Mediation analysis provided evidence for an indirect effect of alcohol on state rumination through concurrent changes in negative mood.

**Conclusions:**

These findings suggest that acute alcohol consumption can regulate negative mood and concurrently rumination, providing preliminary evidence for the role of rumination in alcohol use disorders. Rumination may be a treatment target in alcohol use disorders.

## Introduction

In the UK, harmful alcohol consumption is the leading risk factor for mortality, morbidity and disability among 15–49 year olds (Burton et al. 2016). Considering the burden associated with alcohol misuse, understanding factors involved in the onset and maintenance of, and relapse in alcohol use disorders is crucial for developing new treatments and prevention. One such factor that remains unexplored, and may represent a potential target for treatment, is rumination. According to the Response Styles Theory, rumination is a thinking style that is characterised by a repetitive and passive focus on one’s depressive symptoms, their meaning and implications (Nolen-Hoeksema 1987; Nolen-Hoeksema 1991; Nolen-Hoeksema and Morrow 1993). Although the Response Styles Theory defines rumination in the context of depression, rumination has been subsequently conceptualised as a transdiagnostic process, characterised by repetitive negative thinking that is difficult to control (Ehring and Watkins 2008). Rumination has been causally implicated in the onset and maintenance of depressive and anxiety disorders (Eshun 2000; Ito et al. 2003; Kuehner and Weber 1999; Kuyken et al. 2006; Lam et al. 2003; Papadakis et al. 2006; Richmond et al. 2001; Thomsen 2006), which are often comorbid with alcohol use disorders (Caselli et al. 2013; Grynberg et al. 2016; Hilt et al. 2017; Nolen-Hoeksema and Harrell 2002). Approximately 20% of those with alcohol use disorders are estimated to have concurrent depressive disorders (Grant et al. 2004). In a population with depressive and/or anxiety disorders, rates of alcohol dependence were found to range from 12 to 20% compared to 5% in controls with no anxiety or depressive disorders, with the odds of having alcohol dependence increasing over 4 times among those with both anxiety and depressive disorders (Boschloo et al. 2011). Comorbid alcohol use disorders were more likely to be secondary to the anxiety and depressive disorders (Boschloo et al. 2011) and in longitudinal studies those with primary depressive disorders report subsequent onset of alcohol consumption (Dixit and Crum 2000; Repetto et al. 2004), indicating that those with depressive and anxiety disorders may misuse alcohol to regulate their symptoms.

There is accumulating evidence to support the proposition that rumination is a transdiagnostic process involved in a variety of psychiatric disorders including alcohol and substance use disorders (Ehring and Watkins 2008; Nicolai et al. 2016; Nolen-Hoeksema et al. 2007; Nolen-Hoeksema and Watkins 2011; Smith et al. 2018; Watkins and Roberts 2020). A recent systematic review has demonstrated a strong positive association between types of ruminative thinking and alcohol use or associated problems (Devynck et al. 2019), providing further support to examining rumination as a risk factor and target of treatment within alcohol use disorders. This review also provides support for a transdiagnostic approach, focusing on the processing style of rumination, rather than the disorder specific forms (e.g. depressive rumination in depression, post-event processing in social anxiety) as these were reported to result in the same negative consequences of alcohol misuse (Devynck et al. 2019).

This link between rumination and alcohol use disorders may be mediated by depressive mood given the demonstrated link between rumination and depressive disorders and the concurrence of alcohol use disorders with depression. However, further research is needed to understand mechanisms of the relationship between rumination and alcohol use.

Recently, researchers have examined the impact of rumination and other forms of repetitive negative thinking in alcohol use disorders (Devynck et al. 2019). Firstly, problem drinkers and those with alcohol use disorders have been found to report more frequent use of brooding rumination (negative, self-critical, evaluative thinking), reflective pondering (a form of self-focus with the aim of dealing with problems) (Caselli et al. 2008; Devynck et al. 2017) and abstract/analytical thinking compared to controls, even after adjusting for anxiety and depressive symptoms (Grynberg et al. 2016). Abstract/analytical thinking is a processing style that is characteristic of rumination and involves a high level of construal about the causes, meaning and consequences of experiences (analysing ‘why’ rather than ‘how’) (Watkins 2008; Watkins and Moberly 2009). In cross-sectional studies, there is emerging evidence for a link between alcohol use disorders and rumination. The tendency to ruminate has been found to predict alcohol use as well as seeking treatment for alcohol abuse, above and beyond depression in one study (Caselli et al. 2008), but in another study, this relationship was eliminated when controlling for depression and anxiety symptoms (Devynck et al. 2017). In a prospective study, rumination levels prior to treatment for alcohol abuse predicted drinking status and drinking levels at follow-up when controlling for baseline drinking levels and depression (Caselli et al. 2010).

Whilst studies conducted within a clinical population provided some support for the role for rumination in alcohol use disorders, studies among adolescents and university students from the general population report inconsistent results. Findings in some studies have suggested that increased rumination can predict higher alcohol use (Aldridge-Gerry et al. 2011; Bravo et al. 2018; Hilt et al. 2017) whereas another study reported that brooding rumination predicted lower alcohol use (Willem et al. 2011). On the other hand, a number of studies found no significant relationship between brooding, reflection or depressive rumination and alcohol use (Adrian et al. 2014; Ciesla et al. 2011; Goldstein 2006). Some authors suggest that these contradictory results may be explained by the level of alcohol use (Ciesla et al. 2011; Willem et al. 2014). This is supported by the finding that a rumination induction (Nolen-Hoeksema and Morrow 1991) increased craving for alcohol in individuals with severe alcohol use disorders but not in problem or social drinkers (Caselli et al. 2013). One possibility is that rumination may predict alcohol use in clinical populations but not in the general population, potentially due to higher levels of depressive and anxiety symptoms in those with alcohol use disorders. Indeed, one study demonstrated that the link between repetitive negative thinking and alcohol use disorders was partly mediated by depressive and anxiety symptoms (Devynck et al. 2017). This is in line with the Response Style Theory account of rumination (Nolen-Hoeksema 2000; Nolen-Hoeksema et al. 1999) and with previous literature demonstrating that rumination amplifies existing negative affective states such as depression, anxiety, sad or angry mood; however it does not impact those in a euthymic mood (Watkins and Roberts 2020).

The observed relationship between rumination and alcohol use could reflect different causal directions. Rumination may contribute to the development and maintenance of alcohol use disorders (Caselli et al. 2008; Caselli et al. 2010) by increasing the likelihood of drinking. Rumination is known to exacerbate negative cognitive-affective states (Koval et al. 2012; Lyubomirsky and Nolen-Hoeksema 1995; Nolen-Hoeksema and Morrow 1993; Simons et al. 2017), which in turn can act as triggers for alcohol consumption. According to the emotional cascade model (Selby et al. 2008; Selby and Joiner Jr. 2009) ruminative thinking and negative mood synergistically exacerbate each other in a self-magnifying manner, leading to vicious cycles of rumination and intense levels of negative affect (Selby et al. 2008; Selby and Joiner Jr. 2013). This can often result in compensatory impulsive behaviours to experience transient relief and distraction from rumination and the associated negative emotional cascade (Watkins and Roberts 2020). One such coping strategy is alcohol consumption; a study of repeated momentary assessments found that synergistic effects of negative mood and rumination predicted subsequent impulsive behaviours including binge drinking (Selby et al. 2016). Moreover, another experience sampling study has demonstrated that alcohol consumption can interrupt the persistence of negative affect, therefore temporarily allowing individuals to attend to positive environmental stimuli (Simons et al. 2017). Thus, rumination could exacerbate negative affect, which leads to increased alcohol consumption as a form of self-medication motivated by subsequent reduction in unpleasant stimulus, e.g. negative affect (Brown et al. 1980; Koob and Volkow 2010; Kuntsche et al. 2005). Hence, the vicious cycle of rumination and dysphoric mood may serve to maintain alcohol consumption with the aim of regulating negative mood and ruminative thinking.

In contrast, alcohol consumption could increase rumination by reducing the cognitive control hypothesised to be required to keep rumination in check. ‘Cognitive control’ is defined as the ability to override and suppress processing of information that is no longer relevant or appropriate (Miyake et al. 2000). It is suggested that the inability to disengage from negative cognition (i.e. exert cognitive control) is an important information processing impairment contributing to the tendency to ruminate (De Raedt and Koster 2010; Koster et al. 2011; Whitmer and Banich 2007). In cross-sectional and prospective designs, impaired cognitive control has been demonstrated to increase state rumination in response to stress (De Lissnyder et al. 2012; Koster et al. 2011). Acute alcohol intoxication has been demonstrated to reduce cognitive control when measured using go/no-go tasks or the stop-signal task (Gan et al. 2014; McCarthy et al. 2012; Weafer and Fillmore 2008; Weafer and Fillmore 2012). Given the proposed effects of cognitive control on rumination, acute alcohol consumption may lead to greater rumination via decreasing cognitive control.

The proposition that alcohol may provide an escape from ruminative self-awareness has not been empirically tested. Although cross-sectional and prospective studies provide some support for the role of rumination in alcohol use disorders, these do not allow the exploration of the acute effects of alcohol consumption on rumination. The focus of this study was to investigate the effect of an acute dose of alcohol on state rumination. Here, it is important to distinguish trait rumination, a dispositional ruminative response style, from state rumination, dynamic momentary changes in ruminative thought that fluctuate over time in response to emotional experiences (Moberly and Watkins 2008; Nolen-Hoeksema 1991; Nolen-Hoeksema 2000). Rumination was induced with a task instructing participants to think about unresolved problems (Roberts et al. 2013). This task has been previously demonstrated to produce sustained, spontaneous and involuntary ruminative thinking as opposed to previous methodologies which utilised deliberate and voluntary focus on instructions to ruminate (Nolen-Hoeksema and Morrow 1993; Roberts et al. 2013). Considering the strength of the evidence on the relationship between rumination and negative mood and alcohol’s effects on interrupting unremitting negative mood, one hypothesis is that alcohol consumption would decrease rumination and this effect would be mediated by reductions in negative affect (Armeli et al. 2003; Simons et al. 2017; Swendsen et al. 2000). An alternative account is that alcohol consumption would decrease cognitive control, which in turn would increase rumination, as a result of inability to control ruminative thoughts (De Lissnyder et al. 2012; De Raedt and Koster 2010; Koster et al. 2011; Whitmer and Banich 2007). Examining each of these hypotheses would require a different experimental design. In order to test whether alcohol provides relief from rumination, the rumination induction would need to precede the consumption of alcohol. In contrast, to investigate whether alcohol impairs cognitive control which leads to increased rumination, the reduction of cognitive control i.e. alcohol consumption would need to take place prior to the induction of rumination. Therefore, as only one of these hypotheses can be clearly tested in a single experiment, the current study focuses on the former by inducing rumination first. The measures of cognitive control here are secondary, and this study does not formally test the cognitive control account of the effect of drinking on rumination.

## Methods

### Participants

Participants were recruited from advertisements on social media and in the local area in Exeter. Five hundred forty-five individuals completed the screening questionnaires, of which 169 met the eligibility criteria and 97 eligible participants took part in the study. Six participants dropped out during testing due to feeling sick and one was excluded at the start of the experiment due to reporting severe depression on the Beck Depression Inventory-II (BDI-II) (Beck et al. 1996; Steer et al. 1999) and was given signposting to appropriate services. None of the participants recruited reported active suicidal thoughts which would require exclusion. Ninety participants completed the study.

To be eligible, participants needed to (a) be 18–65 years old, (b) score 8–19 on the Alcohol Use Disorder Identification Test (AUDIT) (Saunders et al. 1993) and (c) score >16 on the Response Styles Questionnaire-Ruminative Response Scale (RRS) short form (Treynor et al. 2003), a measure of trait rumination. Exclusion criteria were current suicidal ideation, severe depression/anxiety measured as respectively as scores of 29 and 26 on the BDI-II and the Beck Anxiety Inventory (BAI) (Beck et al. 1988), presence of schizophrenia and psychosis and taking medications that interact with alcohol. Those with severe depression/anxiety were excluded as rumination has been demonstrated to exacerbate existing negative mood states such as anxiety and depression (Blagden and Craske 1996; Nolen-Hoeksema et al. 2008), therefore the experimental procedure of inducing rumination was expected to cause significant distress in this population.

### Power

As this was a pilot study and there were no previous studies assessing the acute effect on alcohol on ruminative thinking, we did not have any studies to directly base power calculations on. However, Roberts et al. (2013, 2020) reported on the effect of the goal cueing task on state rumination measured as number of thoughts of cued goal during a Sustained Attention Reaction Time Task. On the basis of this study, based on an effect size of *d* = 0.87, to achieve a power of 95%, we would need 30 participants per group.

### Design

The study employed a double-blind randomised placebo-controlled design. The experiment involved inducing rumination by asking participants to focus on an important ongoing concern or problem. Following the rumination induction, participants were randomly assigned to one of three groups (placebo, low alcohol dose (0.4 g kg^−1^), or high alcohol dose (0.8 g kg^−1^)) to assess the effect of alcohol consumption on affect, rumination and cognitive control. The study was approved by the University of Exeter ethics committee.

### Alcohol administration

The volume of dose (*D*) consumed was calculated as *D* = *W*_*ρ*_ (*C*_*t*_ *+ β*_*t*_), where *W* was body weight in kg, *ρ* was the volume of distribution of alcohol in the body (l kg^−1^), *C*_*t*_ the BAC (g 100 ml^−1^) at time *t*, *β* the elimination rate and *t* the time (h) from dose. Based on the work of Gullberg and Jones (1994), we set *β* to 0.015 g 100 ml^−1^ h^−1^ and following Friel et al. (1995), we set *ρ* values to 0.71 and 0.65 for males and females, respectively. Alcohol groups consumed ethanol combined with tonic water, and tabasco which was found to mask the group allocation in pilot work.

Participants were blinded to the alcohol condition they were assigned to and were told that they would be given drinks which may or may not contain alcohol. An unblinded researcher determined allocation from randomisation codes kept in sealed envelopes and prepared the drinks for administration. Participants randomised to low and high dose alcoholic drink conditions received in total 0.4 g kg^−1^ and 0.8 g kg^−1^ of alcohol, respectively. The drinks were divided into six small cups, and each cup contained 150 ml of liquid, including the alcohol dose, tonic water and five drops of tabasco. Those in the placebo condition were given the same amount of liquid consisting of tonic water and five drops of tabasco. All participants were given two small cups of drinks (2 * 150 ml) to drink in approximately 10 min. The same procedure was repeated until all six cups were consumed by the participants. After the participants finished the first drinks, unblinded researchers who were not involved with data collection took breathalyser readings every 10 min for an hour.

### Experimental tasks

#### Unresolved goal cueing task

The unresolved goal cueing task (Roberts et al. 2013) was used to induce rumination. This task has previously been found to induce state rumination that persisted for 40 min through a cognitive task (Roberts et al. 2013), and thus was deemed suitable to investigate the short-term effect of drinking on rumination. The participants were asked to identify an ongoing or unresolved concern that they repeatedly thought about and that led to negative feelings. Example topics included ‘an ongoing concern about an important relationship’. Participants then wrote down the unresolved goal and evaluated the goal using a number of scales including the importance of the problem and the level of distress it caused (rated on a scale from not at all (0) to very much (10)). During the next 10 min, participants were guided through focusing on their unresolved goal, for instance ‘think about what is important about this difficulty in terms of your personal goals’ and ‘focus on how this problem reflects a lack of progress on important personal goals’. These instructions were delivered on a pre-recorded audio script over headphones. This task was demonstrated to lead to increased number of thoughts of cued goal up to 50 min after the task, compared to cueing a resolved goal (Roberts et al. 2013; Roberts et al. 2020).

#### Stop-signal task

The stop-signal task was used to tap cognitive control (Logan et al. 1984; Verbruggen and Logan 2008). All stimuli were presented on a 24-in. LED monitor against a white background. The task was run using the Psychtoolbox and Matlab (Brainard 1997). The stimuli consisted of a green arrow (go stimuli) and a red arrow (stop signal). Participants were told that an arrow would appear on the screen every few seconds, pointing to either left or right. They were asked to respond as fast as possible with their left and right index fingers to indicate the direction of the arrow, using the keys C and M, respectively. Participants were told that the green arrow would be followed by a red arrow on some trials, in which case they should not press any buttons and wait for the next arrow to appear. No-signal trials started with the presentation of a fixation cross, after which the green arrow appeared on the screen. The green arrow remained on the screen for 500 ms or until the participants had responded. On stop-signal trials, the green arrow turned red after a variable stop-signal delay (stimulus-onset asynchrony (SOA)). The SOA was dynamically adjusted using a tracking procedure, which ensured that each participant would succeed in withholding their response on approximately 50% of the stop-signal trials. When participants successfully stopped their response, this delay was decreased by 50 ms on the following trial, which made it harder to successfully stop. When participants failed to stop in time, the delay was increased by 50 ms. The task consisted of three blocks and participants had 10 s break between each block. There were a total of 72 trials per block, with 54 go and 18 stop-signal trials. There were no practice blocks. The index of cognitive control in this task is stop-signal reaction time (Verbruggen and Logan 2008).

### Questionnaires

#### AUDIT

AUDIT is a ten-item self-report questionnaire to provide an index level of alcohol use disorder (Saunders et al. 1993). Items are rated on a five point scale (0–4). Hazardous drinking (score of 8–15 on the AUDIT) is defined as a pattern of drinking that makes individuals increasingly vulnerable to harmful consequences of alcohol use. A pattern of alcohol consumption that results in consequences to physical and mental health is considered harmful drinking (scores of 16–19 on the AUDIT).

#### Drinking Motives Questionnaire-Revised

Drinking Motives Questionnaire-Revised (DMQ-R) is a 20-item tool that measures individuals’ score on four types of motives for drinking (Cooper 1994; Kuntsche et al. 2006). These include social motives—drinking to be sociable, coping motives—drinking to cope with negative mood, enhancement motives— drinking to enhance situations and conformity motives—drinking to fit in with others.

#### Biphasic Alcohol Effects Scale

Biphasic Alcohol Effects Scale (BAES) is a-14 item adjective rating scale which measures the stimulating and sedative effects of acute alcohol consumption (Martin et al. 1993). Items measuring stimulating effects include talkative, up and vigorous; whereas, the sedative effects of alcohol are measured by items such as inactive, heavy head and slow thoughts.

#### BAI

BAI is a 21-item self-report questionnaire to measure severity of anxiety symptoms during the past 2 weeks, including difficulty breathing, feeling nervous or scared (Beck et al. 1988). Items are rated on a 4-point scale (0–3) with higher scores indicating more severe symptoms. Scores <8 indicate minimal anxiety, 8–15 mild anxiety, 16–25 moderate anxiety and 26+ severe anxiety.

#### BDI-II

BDI-II is a twenty-one-item self-report questionnaire to measure severity of depressive symptoms during the past 2 weeks including sadness, loss of pleasure and self-criticism (Beck et al. 1996; Steer et al. 1999). Items are rated on a 4-point scale (0 to 3), with higher scores indicating more severe symptoms. Scores <13 indicate absence of depression, 14–19 mild depression, 20–28 moderate depression and 29–63 severe depression.

#### Bond and Lader Visual Analogue Scales

These consists of 16 items measuring subjective feelings including: alertness, attentiveness, contentedness, calmness and happiness on a Visual Analogue Scale (VAS) (Bond and Lader 1974). Factor analysis yields three factors: alertness, negative affect and calmness.

##### Negative affect

Items relating to negative affect within the Bond and Lader VAS were discontented, troubled, sad, antagonistic and withdrawn. Higher scores indicate increased negative affect.

#### Ruminative Thought Style Questionnaire

Ruminative Thought Style Questionnaire (RTSQ) is a twenty-item questionnaire which measures positive, negative and neutral ruminative thoughts (Brinker and Dozois 2009). Items are scored on a 7-point scale from (1–7) higher scores indicating more ruminative thinking. Four factors are extracted from the RTSQ: problem focused thoughts, counterfactual thinking, repetitive thoughts and anticipatory thoughts (Tanner et al. 2013).

#### RRS-short form

RRS-short form includes 10 items measuring the frequency of ruminative response, consisting of two factors: brooding and reflection (Treynor et al. 2003). Items are rated on a 4-point scale from 1 (almost never) to 4 (almost always). Brooding refers to passive comparison of one’s situation with unachieved standards and is considered to be a pathological form of rumination, whereas reflection involves self-focus with the aim of dealing with depressive symptoms and is considered to be an adaptive coping strategy.

#### Brief State Rumination Inventory

Brief State Rumination Inventory (BSRI) is an eight-item measure of state rumination assessed on a VAS from strongly disagree (0 mm) to strongly agree (100 mm) (Marchetti et al. 2018). Items include ‘Right now, it’s hard for me to shut off negative feelings about myself’. The BSRI has been demonstrated to have good psychometric properties in terms of reliability, a stable factor structure and validity (Marchetti et al. 2018). Additionally, it was found to be sensitive to the experimental manipulation of rumination (Marchetti et al. 2018). We calculated the Cronbach’s alpha for the BSRI in the current study using baseline ratings, *α* = 0.834 indicated high level of internal consistency of BSRI items.

#### State rumination about the unresolved goal

In the present study, an additional item was included in the BSRI to measure state rumination about the unresolved goal: ‘Right now, I am thinking about the problem, goal or concern identified in the task earlier’.

### Procedure

**Fig. 1:**
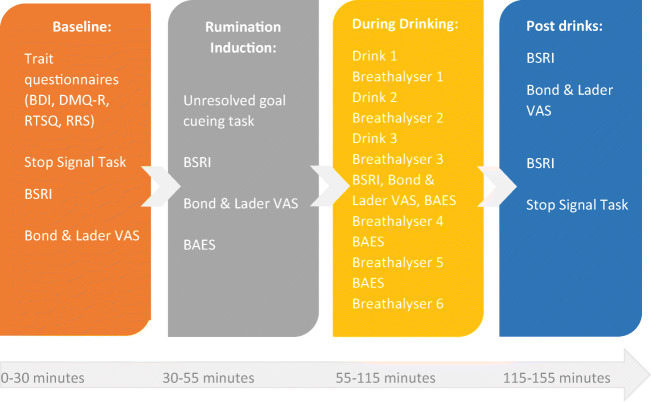
Study procedures with approximate timings

Interested participants were invited to contact researchers to request an online screening questionnaire. Eligible participants were notified and a testing day was arranged. Upon arrival to the laboratory, participants gave written and witnessed informed consent. Prior to starting the experiment, participants gave a breathalyser reading and had their weight recorded to determine the alcohol volume. We also administered the BDI-II to check for current suicidal ideation and severe depression scores, and appropriate risk protocols were followed where required. Participants completed the questionnaires and tasks in the order shown in Fig. 1. At the end of the laboratory experiment, participants were given a positive mood induction and were reimbursed for their time.

### Analysis

The statistical analyses were completed on IBM SPSS.25. All statistical tests were two tailed. Assumptions of repeated measures ANOVA and between subjects ANOVA were checked prior to analyses. We identified outliers for the stop-signal reaction times (<50 and negative scores) (Congdon et al. 2012) at baseline and post-drinks. These were excluded from the analysis of the relevant dependent variables (DV). Where the DVs did not meet the assumptions of normality using Shapiro-Wilks test, we used exponential transformation for negatively skewed data and logarithmic transformation for positively skewed data. However, neither transformation resulted in normal distribution of the data; therefore, we analysed the untransformed data. Sphericity assumptions were checked on the SPSS output; where sphericity was violated, we reported the Huynh-Feldt correction. Bonferroni corrections were used for multiple comparisons.

We conducted a series of Kruskal-Wallis tests for non-parametric data and one-way ANOVAs for parametric data (or Chi-square test where applicable), with condition as independent variable (IV), and baseline demographic, alcohol use and mental health characteristic variables as DV. We also conducted mixed methods ANOVA with time as repeated measures IV (baseline, 20 min, 30 min, 40 min, 50 min and 60 min after first drink), condition (placebo, low-dose or high-dose alcohol) as between subjects IV and breathalyser readings as DV to compare breath alcohol concentrations across the groups.

To investigate our main hypothesis, a mixed ANOVA with time as within-subject IV (baseline, post-rumination induction, 30 min, 60 min and 90 min after first drink), condition (placebo, low-dose and high-dose alcohol) as between subjects IV and state rumination about the unresolved goal as DV was performed. We then repeated the same analysis with the total state rumination score (BSRI). Following this, we conducted mixed measures ANOVA with time as within-subject IV (baseline, post-induction, 30 min, 60 min), condition (placebo, low-dose and high-dose alcohol) as between-subject IV and negative mood as DV. Mediation analysis was performed using the PROCESS version 3.4 macro for SPSS (Hayes 2012). We conducted a mediation analysis, using model 4, with number of bootstrapping samples set to 5000 and with confidence intervals of 95%. Our independent variable (*X*) was alcohol dose (0, 0.4 g kg^−1^, 0.8 g kg^−1^), the mediator was negative affect (*M*) and the outcome was state rumination (*Y*). To detect the effects of alcohol on sedation and stimulation, we conducted a series of mixed ANOVAs with time as within-subject IV (baseline, 30 min, 40 min and 50 min after first drink), condition as between-subject IV and sedation and stimulation as DVs, respectively.

As an exploratory analysis, a mixed ANOVA with condition as between-subject IV and time as within-subject IV (baseline and after drinks administration) was conducted on stop-signal reaction time. We also performed Pearson’s and Spearman’s correlations between measures of rumination and cognitive control.

## Results

### Participants

The three groups were matched well on demographic characteristics with no significant differences in gender, age or ethnicity distribution across the groups (see Table 1). The overall mean AUDIT score was 11.61 (SD = 3.06), indicating a hazardous level of drinking (scores from 8 to 19). There were no statistically significant differences in AUDIT scores nor any of the drinking motives between the three conditions. Additionally, there were no statistically significant differences in Brooding subscale of the RRS short from nor in any of the four factors of RTSQ. However, there was a statistically significant difference between the groups in Reflection subscale of the RRS short form, with the low-dose alcohol group reporting lower mean reflection scores compared to both placebo (*p* = 0.033) and high-dose alcohol group (0.027). There were also no significant differences among the groups regarding total depression and anxiety scores as measured by BDI-II and BAI, respectively, with overall mean scores (BDI-II: 10.91 (SD = 6.65), BAI: 8.76 (SD = 6.47)), indicating minimal levels of depression and anxiety.

**Table 1:** Baseline characteristics including demographics, alcohol use and mental health across groups

	Placebo (*n* = 31)	Low-dose alcohol (*n* = 30)	High-dose alcohol (*n* = 29)	Test statistics	*p* value
Gender (*N*)	11 males 20 females	11 males 19 females	12 males 17 females	*X*^2^(2) = 0.25	0.885
Age (*M* (SD))^a^	25.13 (12.16)	22.14 (5.4)	22.90 (7.68)	*F*(2, 85) = 0.9	0.409
Ethnicity (*N*)	25 White^a^ 6 others	20 White^a^ 10 others	24 White^a^ 5 others	*X*^2^(2) = 8.52	0.385
AUDIT (Mdn (IQR))	11 (4)	13 (6)	10 (6)	*F*(2, 87) = 2.76	0.252
DMQ-R subscale scores					
Drinking to cope (Mdn (IQR))	2 (1.20)	2.2 (1.2)	2 (0.8)	*X*^2^(2) = 0.003	0.999
Drinking for enhancement (M (SD))	3.18 (0.79)	3.37 (0.8)	3.05 (0.67)	*F* (2, 87) = 1.38	0.258
Drinking for socialisation (Mdn (IQR))	3.8 (1.2)	3.8 (1.45)	3.6 (1.1)	*X*^2^(2) = 0.19	0.911
Drinking for conformity (Mdn (IQR))	1.6 (1.4)	1.7 (1.05)	1.4 (1)	*X*^2^(2) = 1.83	0.400
Brooding (M (SD))	9.7 (2.65)	10.83 (3.27)	10.66 (2.89)	*F*(2, 87) = 1.28	0.282
Reflection (M (SD))	11.52 (2.92)	9.83 (2.89)	11.62 (3.31)	*F* (2, 87) = 3.26	0.043
Problem-focused thinking (M (SD))	15.07 (7.07)	14.6 (6.43)	15.67 (5.67)	*F*(2, 87) = 0.23	0.795
Counterfactual thinking (M (SD))	17.19 (6.47)	16.8 (5.49)	17.41 (5)	*F*(2, 87) = 0.09	0.916
Repetitive thoughts (M (SD))	18.19 (5.08)	18.83 (5.23)	19.33 (4.68)	*F*(2, 87) = 0.38	0.687
Anticipatory thinking (Mdn (IQR))	7 (7)	7.5 (5)	8 (3)	*X*^2^(2) = 1.65	0.438
BDI-II (Mdn (IQR))	11 (7)	10 (8.5)	9 (6.5)	*X*^2^(2) = 1.05	0.591
BAI (Mdn (IQR))	8 (9)	7.5 (7.5)	7 (10.5)	*X*^2^(2) = 0.12	0.943

Note: *AUDIT*, Alcohol Use Disorders Identification Test; *BDI-II*, Beck Depression Inventory II; *BAI*, Beck Anxiety Inventory; *DMQ-R*, Drinking Motives Questionnaire-Revised; *M*, mean; *SD*, standard deviation; *Mdn*, median; *IQR*, interquartile range; *N*, number. ^a^Includes White UK & European) a: 2 participants had missing data for the age variable

### Manipulation check

#### Breath-alcohol concentrations across time and condition

**Fig. 2:**
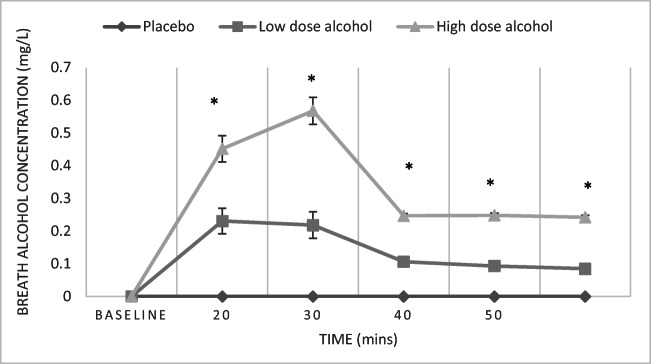
Mean BRAC (mg l^−1^) across time of testing and alcohol administration group. There were significant (*p < 0.05) group differences in BRAC at all time points except at baseline. Error bars represent standard error of the mean. Thirty-one participants contributed to placebo group, 30 to low-dose alcohol group and 29 to high-dose alcohol group

There was a significant time by condition interaction on breath-alcohol concentrations (BRAC) [*F*(4.31, 187.27) = 20.23, *p* < 0.001, *η*_p_^2^ = 0.32] with significant differences between the groups at all time points except baseline (see Fig. 2). Condition [*F*(2, 87) = 142.23, *p* < 0.001, *η*_p_^2^ = 0.77] and time also had significant main effects on BRAC [*F*(2.15, 187.27) = 54.51, *p* < 001, *η*_p_^2^ = 0.39].

### State rumination about the unresolved problem

**Fig. 3:**
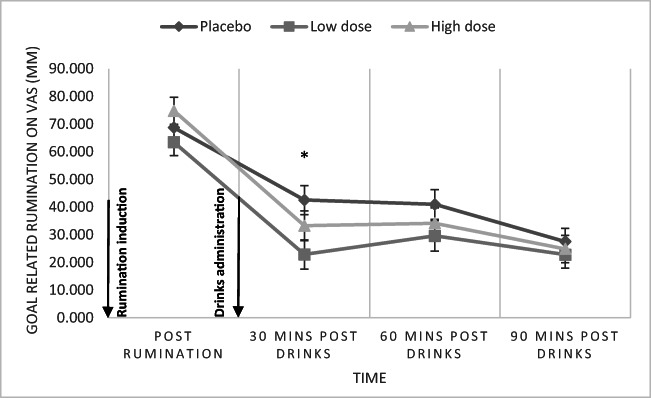
Mean state rumination about an unresolved problem over time and groups (low- and high-dose alcohol conditions collapsed together). There was a significant difference in state rumination between the placebo and the low-dose alcohol group at 30 min post-alcohol administration (*p < 0.05). Error bars represent standard error of the mean. 31 participants contributed to placebo group, 30 to low-dose alcohol group and 29 to high-dose alcohol group

The interaction of condition by time on state rumination was not statistically significant [*F*(5.31, 231.07) = 1.77, *p* = 0.116, *η*_p_^2^ = 0.04]. Though Bonferroni-corrected simple effects analysis revealed a significant reduction in rumination about the unresolved problem in the low alcohol condition when compared to placebo (*p* = 0.029), 30 min after participants had their first drink (see Fig. 3). However, there was no significant difference in state rumination between the high alcohol and placebo conditions at this time point (*p* = 0.657). There was a significant main effect of time on rumination about the unresolved problem [*F*(2.66, 231.07) = 104.87, *p* < 0.001, *η*_p_^2^ = 0.55] but not of condition [*F*(2, 87) = 1.54, *p* = 0.219, *η*_p_^2^ = 0.03].

### State rumination measured by total BSRI

**Fig. 4:**
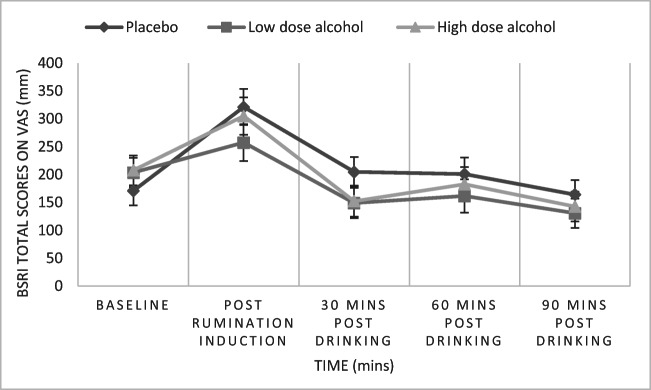
Mean state rumination measured using BSRI total scores over time and groups. Time*Condition interaction was not statistically significant. Error bars represent standard error of the mean. Respectively, 30 and 29 participants contributed data to low-dose and high-dose alcohol groups, and 31 contributed data to placebo group

There was a significant main effect of time on total BSRI scores [*F*(3.1, 269.91) = 39.67, *p* < 0.001, *η*_p_^2^ = 0.31]. Pairwise comparisons revealed that BSRI scores significantly increased following the goal cueing task compared to baseline (*p* < 0.001). There was a significant decrease 30 min after drink administration (*p* < 0.001). Although there was not a significant difference between 30 and 60 min following drink administration (*p* = 0.186), at 90 min post-drink a significant decrease in BSRI scores was observed compared to 60 min (*p* < 0.001). We did not find a significant main effect of condition [*F*(2, 87) = 0.42, *p* = 0.660, *η*_p_^2^ = 0.01], nor a significant time and condition interaction [*F*(6.21, 269.91) = 1.91, *p* = 0.076, *η*_p_^2^ = 0.04] on total BSRI scores (see Fig. 4).

### Negative mood measured by the Bond and Lader VAS

Time had a significant main effect on the factor of negative mood [*F*(2.77, 240.57) = 37.77, *p* < 0.001, *η*_p_^2^ = 0.3]. Pairwise comparisons revealed that there was a significant increase in negative affect following the goal cueing induction (*p* < 0.001), and this significantly decreased at 30 post-first drink (*p* < 0.001), but not at 60 min following the first drink (*p* = 1.0). There was no main effect of condition on negative affect [*F*(2, 87) = 2.24, *p* = 0.112, *η*_p_^2^ = 0.05]. There was no significant interaction of time and condition on negative affect either [*F*(5.53, 240.57) = 1.14, *p* = 0.340, *η*_p_^2^ = 0.03].

### Biphasic effects of alcohol across group and time

There was a significant main effect of time on sedation [*F*(1.24, 105.45) = 12.93, *p* < 0.001, *η*_p_^2^ = 0.13] (Table 2). According to the pairwise comparisons, there was a significant reduction in sedation between baseline and 30 min after the first drink (*p* < 0.001), followed by a significant increase in sedation between 30 and 40 min after the first drink (*p* = 0.001), and no significant difference in sedation between 40 and 50 min following participants’ first drink (*p* = 0.123). Condition also had a significant main effect on sedation [*F*(2, 85) = 9.14, *p* < 0.001, *η*_p_^2^ = 0.18], with higher sedation scores in the high alcohol group compared to both low alcohol (*p* = 0.001), and placebo condition (*p* < 0.001). We did not observe a statistically significant interaction of time and condition on sedation [*F*(2.48, 105.45) = 2.04, *p* = 0.125, *η*_p_^2^ = 0.05].

**Table 2:** Biphasic effects of alcohol over time and across groups (M (SD))

	Placebo^b^	Low alcohol dose^b^	High alcohol dose^b^
*Sedation*			
Baseline^a^	43.68 (37.65)	29.61 (34.95)	53.48 (34.96)
30 min post-drinks^a^	18.68 (10.87)	24.79 (11.42)	26.31 (12.4)
40 min post-drinks^a^	22.19 (13.11)	27.21 (16.27)	35.48 (12.89)
50 min post-drinks^a^	21.32 (13.98)	27.21 (18.38)	33.38 (12.71)
*Stimulation*			
Baseline	22.8 (15.49)	21.59 (13.54)	22.52 (12.19)
30 min post-drinks	24.68 (14.9)	20.9 (15.59)	25.11 (14.16)
40 min post-drinks	24.17 (13.13)	20.62 (15.06)	26.11 (13.97)
50 min post-drinks	23.13 (13.85)	20.66 (13.76)	26.07 (13.78)

Note: *M*, mean; *SD*, standard deviation. ^a^Time had a significant main effect on sedation, with significant differences in sedation from baseline to 30 min post-drinks (*p* < 0.001) and from 30 to 40 min post-drinks (*p* = 0.001). ^b^Condition had a significant main effect on sedation, with significant differences between high dose and low dose (*p* = 0.001) and high dose and placebo (*p* < 0.001). For sedation, 31 participants contributed to the placebo group, 28 to the low dose alcohol group and 29 to the high dose alcohol group. For stimulation, 30 participants contributed to the placebo group, 29 to the low dose alcohol group and 27 to the high dose alcohol group

We did not find any main effects of time [*F*(1.71, 141.55) = 0.46, *p* = 0.604, *η*_p_^2^ = 0.01], condition [*F*(2, 83) = 0.81, *p* = −0.449, *η*_p_^2^ = 0.02] nor a time * condition interaction no interaction on stimulation [*F*(3.41, 141.55) = 0.53, *p* = 0.685, *η*_p_^2^ = 0.01].

### Exploring the mediation of the effect of alcohol on rumination by negative mood

**Fig. 5:**
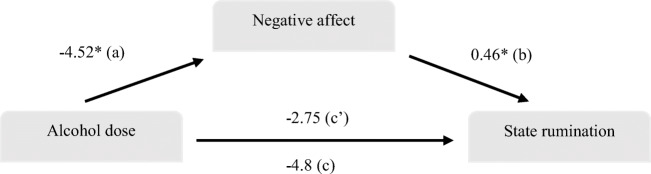
Mediation model demonstrating the indirect effect of alcohol dose on rumination by change in negative affect ratings. Significant effects are denoted by an asterisk

We conducted a mediation analysis with alcohol dose (0, 0.4 g kg^−1^, 0.8 g kg^−1^) as the predictor, negative affect as the mediator and rumination about the unresolved goal as the outcome, both measured at 30 min after participants consumed their first drink. The mediation analysis suggested that alcohol dose was a significant predictor of the mediator variable negative affect, *b* = −4.52, *t* (88) = −2.18, *p* = 0.03 (a pathway), the mediator negative affect was also a significant predictor of the outcome variable rumination, *b* = 0.46, *t* (87) = 2.38, *p* = 0.02 (*b* pathway). There was no significant total effect of *X* on *Y*, *b* = −4.82, *t* (88) = −1.25, *p* = 0.214 (*c* pathway) nor a direct effect of *X* on *Y*, *b* = −2.75, *t* (88) = −0.71, *p* = 0.477 (*c*’ pathway) (see Fig. 5). The effect size for the indirect effects of *X* (alcohol dose) on *Y* (rumination) through *M* (negative affect) was *b* = −2.07 (95% confidence interval: −4.77 to −0.15). As the 95% confidence interval for the indirect effect does not contain 0, we can reject the null hypothesis that the true indirect effect is 0 at 0.05 level of significance (See Hayes (2009) for explanation of indirect effects in the absence of total effect).

### Exploratory analyses

#### Stop-signal reaction time

There was a significant main effect of time on stop-signal reaction time [*F*(1, 76) = 7.73, *p* = 0.007, *η*_p_^2^ = 0.09], with the reaction time increasing over time in all participants (Table 3). We did not find a significant main effect of condition [*F*(2, 76) = 1.45, *p* = 0.241, *η*_p_^2^ = 0.04] nor a significant time * condition interaction [*F*(2, 76) = 0.5, *p* = 0.609, *η*_p_^2^ = 0.01].

**Table 3:** Stop-signal reaction time across time and condition *M* (SD)

	Placebo (*n* = 27)^a^	Low-dose alcohol (*n* = 26)^a^	High-dose alcohol (*n* = 26)^a^
Baseline	196.56 (66.72)	198.24 (56.12)	221.38 (45.65)
Post-drinking	218.5 (55.29)	227.54 (46.53)	232.42 (50.48)

Note: *M*, mean; *SD*, standard deviation; *IQR*, Interquartile range. ^a^In the placebo, low-dose, and high-dose groups respectively 4, 4 and 3 participants’ reaction time data was excluded from the analyses due to not meeting assumptions of the stop-signal task (i.e. probability of stopping a response < 30% or >70%) or for being outliers (<50 ms or negative stop-signal reaction time according to Congdon et al. 2012)

### Cognitive control and trait/state rumination

In order to examine the relationship between rumination and baseline levels of cognitive control, we ran a number of Spearman’s (BSRI) and Pearson’s (RTSQ, RRS short-form Brooding and Reflection) correlations with state and trait rumination, and baseline stop-signal reaction time. We did not find any evidence of a correlation between baseline stop-signal reaction time and reflective rumination, *r* (82) = −0.06, *p* = 0.578, nor with Brooding rumination, *r* (82) = 0.03, *p* = 0.799, nor with RTSQ, *r* (82) = 0.15, *p* = 0.175, nor with state rumination at baseline, *r* (82) = −0.1, *p* = 0.396, nor with state rumination at post-rumination induction, *r* (82) = −0.08, *p* = 0.497.

## Discussion

This study set out to examine the effects of acute alcohol consumption on ruminative thinking among a hazardous drinking population. The main finding was lower state rumination about an unresolved problem following a rumination induction in the low-dose alcohol group (0.4 g kg^−1^) at 30 min post-administration when compared to placebo. We also found evidence for an indirect effect of alcohol on rumination through negative affect, whereby alcohol concurrently reduced negative affect ratings and rumination. Additionally, we found a significant increase in state rumination following the rumination induction compared to baseline, therefore indicating that our manipulation was successful. However, we did not observe an effect of alcohol consumption on the overall state rumination compared to placebo. Contrary to the existing literature, the study found no difference in response inhibition when measured using a stop-signal task between the low- and high-dose alcohol and placebo groups. There was also no significant correlation between state and trait rumination and baseline levels of cognitive control as measured by the stop-signal reaction time.

The main finding of this study was that acute low-dose alcohol consumption reduced rumination about an unresolved, personally important problem. During the rumination induction task, participants were specifically instructed to focus on the personal implications of the actual-self discrepancy with prompts such as ‘focus on how the problem reflects a lack of progress on important personal goals’. Therefore, the finding that alcohol reduced ruminative, self-focused thinking about an unresolved goal is in line with previous findings demonstrating that alcohol consumption reduces self-consciousness as well as negative self-relevant thoughts during post-event processing, a type of repetitive negative thinking style involving re-evaluation of one’s behaviour during a recent social interaction (Abrams et al. 2002; Hull 1981; Hull et al. 1983; Hull et al. 1986).

The results of our mediation analysis also support previous studies demonstrating that acute alcohol consumption reduces negative affect (Armeli et al. 2003; Simons et al. 2017; Swendsen et al. 2000) and concurrently state rumination. This study found that the effect of alcohol on ruminative thinking was mediated by concurrent reductions in negative mood. This adds to previous research which demonstrated that the link between repetitive negative thinking (brooding, reflection rumination, abstract-analytical thinking) and alcohol use was mediated by depressive symptoms including negative mood among patients with alcohol use disorders (Devynck et al. 2017), although in our sample depressive symptoms were minimal. It is important to note that the mediation analysis included state rumination and negative mood measures taken at the same time point, therefore we cannot exclude the possibility that reduced rumination as a result of alcohol consumption led to decrease negative mood instead. Nevertheless, the current findings are in line with self-medication theories of alcohol use, which propose that alcohol consumption is motivated by the desire to suppress unpleasant emotions and experiences (Brown et al. 1980; Koob and Volkow 2010; Kuntsche et al. 2005). Whilst not investigated in the current study, our findings are complimentary to those that suggest that alcohol consumption may be motivated by alcohol’s ability to disrupt the negative affect and rumination cycle or emotional inertia (Fairbairn and Sayette 2013; Simons et al. 2017). Future studies can examine the effect of alcohol intake on emotional cascades using momentary assessments of mood, rumination and drinking.

The finding that alcohol consumption can transiently reduce negative mood and consequently rumination has implications for our understanding of factors contributing to the onset and maintenance of alcohol use disorders. Individuals with increased tendency to engage in rumination may be at increased risk of using alcohol as an impulsive and maladaptive strategy to cope with the rumination-induced emotional cascades (Selby et al. 2008; Selby and Joiner 2013; Selby et al. 2016; Watkins and Roberts 2020), since alcohol may provide temporary relief from such mood as demonstrated in this study (Armeli et al. 2003; Simons et al. 2017; Swendsen et al. 2000). Additionally, during recovery, the tendency to ruminate may increase one’s risk of relapsing to alcohol use, as worsening mood is a risk factor for relapse among patients with alcohol use disorders (Driessen et al. 2001; Kushner et al. 2005; Tomasson and Vaglum 1996). These findings suggest that targeting ruminative processes may be particularly helpful for those at risk of developing alcohol use disorders as well as for those in recovery.

Although the present design was not able to test the hypothesis that alcohol consumption may lead to increased rumination through impaired cognitive control, we examined these as exploratory outcomes. There was no evidence in the current study of the predicted impairment in inhibitory control following alcohol administration compared to placebo; this is in contrast with previous research which reported that alcohol consumption resulted in impaired cognitive control (Gan et al. 2014; McCarthy et al. 2012; Weafer and Fillmore 2008; Weafer and Fillmore 2012). This effect could be explained by the population of drinkers recruited to this study, i.e. hazardous/harmful drinkers but not drinkers with severe alcohol use disorders. Previous research suggests that heavy drinkers may be more sensitive to alcohol’s effects on cognitive tasks than lighter drinkers (Field et al. 2007; Perry and Carroll 2008; Petry 2001; Vuchinich and Simpson 1998), whereby increased sensitivity to the impairing effect alcohol was associated with self-reported levels of drinking and heavy drinking (Roberts et al. 2014; Weafer and Fillmore 2015). These findings provide a possible explanation of absence of alcohol induced effects on the stop-signal task in a non-clinical population in this study. Additionally, the second stop-signal task was completed towards the end of a two and half hour long study and the reaction time increased in all participants across the task. Therefore, all participants may have been experiencing the effects of fatigue, and this may have masked the effects of alcohol.

Our exploratory analyses also did not show any relationship between state or trait rumination and cognitive control at baseline, contradicting previous research which demonstrated that impaired cognitive control at baseline was associated with increased brooding rumination following a stressful event (De Lissnyder et al. 2012). The differences in measurement of cognitive control may explain the discrepancy of our findings with the existing research. In the present study, this involved measuring cognitive control for external stimuli, i.e. arrows presented on the screen, whilst De Lissnyder et al. (2012) examined cognitive control ability for internal mental representations held in working memory using an Internal Switch Task (De Lissnyder et al. 2012). Examining cognitive control for internally held stimuli may be more appropriate to understand the link between rumination and cognitive control, as rumination involves repetitive focus on *internal* negative thoughts. Future research aiming to understand whether alcohol’s effects on rumination may be mediated by cognitive control could use tasks designed to measure cognitive control of internal stimuli.

Contrary to our findings in rumination regarding an unresolved goal, we failed to demonstrate an effect of alcohol on overall state rumination using the BSRI. Although our results demonstrate an increase in overall state rumination immediately after the rumination induction, with time this effect quickly dissipates, suggesting that the rumination induction does not have lasting effects on overall state rumination beyond 30 min. The rumination induction may also be more effective at inducing rumination about the unresolved goal, rather than a general state of rumination, as it focuses particularly on the latter. However, it is worth mentioning that the BSRI is a new measure of state rumination (Marchetti et al. 2018) and its sensitivity to interventions to reduce rumination (e.g. alcohol) has not been previously reported. There are limited measures of state rumination, as most research has focused on quantifying general tendency to ruminate (trait), rather than rumination in response to specific situations (state). Moreover, most currently available measures of state rumination involve ‘off-line’ measurement, i.e. assessment of behaviour after it happened (Cladder-Micus et al. 2019). In contrast, ‘on-line’ measurements involve assessing behaviour during task performance, for instance ‘thinking-aloud protocols’ (Cladder-Micus et al. 2019). The Breathing-Focus Task (BFT) (Borkovec et al. 1983; Southworth et al. 2017) is an on-line task during which participants are instructed to focus their attention on their breathing and report if their mind wanders to repetitive negative thoughts (Southworth et al. 2017). The use of on-line measures reduces retrospective biases and response bias (Cladder-Micus et al. 2019) and may prove to be a more sensitive measure of the effects of alcohol on state rumination.

### Strengths and limitations

We found that the average breath alcohol concentration has reached a steady state at 40 min following the first drink administration. An additional strength of the current study was the robust increases in overall state rumination observed following our rumination induction method. However, we did not have a pre-rumination induction baseline measure of goal-related state rumination to confirm that the rumination induction was successful, although participants have reported high levels of agreement with the BSRI goal-related item at post-rumination induction.

A limitation of this study was that we were only able to include hazardous and harmful drinking populations due to ethical concerns associated with giving alcohol to individuals with alcohol use disorders. This may be a limitation as previous studies have found support for the role of rumination in drinking for clinical populations (Caselli et al. 2008; Devynck et al. 2017; Devynck et al. 2019; Grynberg et al. 2016); however, findings were mixed among the general population (Adrian et al. 2014; Aldridge-Gerry et al. 2011; Bravo et al. 2018; Ciesla et al. 2011; Goldstein 2006; Hilt et al. 2017; Willem et al. 2011). Nonetheless, our results show preliminary evidence for a relationship between alcohol use and rumination even in non-clinical populations.

Another limitation was that we excluded individuals with severe depression and severe anxiety due to concerns about using the rumination induction in this group. Our sample reported mild levels of anxiety and depression on average. Considering trait rumination is linked to the severity of depression and/or anxiety disorders (Nolen-Hoeksema 2000), it is likely that we were not able to include a sample of participants with the highest levels of trait rumination. Indeed, on average, our sample reported ruminating ‘sometimes’ in response to negative mood, but not ‘often’ or ‘always’. Additionally, due to the design constraints, we were only able to examine one direction of impact of alcohol on rumination, i.e. whether alcohol consumption would reduce rumination through its impact on mood. The alternative hypothesis on whether alcohol impairs cognitive control and subsequently increases state rumination remains to be explored. Finally, we did not find the expected biphasic alcohol effects on sedation and stimulation over time and across conditions (Martin et al. 1993; Rueger et al. 2009).

### Conclusions

This study set out to explore how alcohol consumption acutely impacts state rumination, following a rumination induction. We found evidence for a reduction in goal-related state rumination following low-dose alcohol consumption but not in general state rumination. The mediation analysis supported alcohol’s ability to regulate rumination through concurrent reductions in negative mood; therefore, the study provides preliminary evidence for rumination as a factor involved in the onset and maintenance of alcohol use disorders. Targeting ruminative thinking patterns may be promising new avenue for treatment of alcohol use disorders.

## Declarations

### Data access statement

The research data supporting this publication will be openly available from the University of Exeter's institutional repository upon publication.
